# Predicting unfavorable long-term outcome in juvenile idiopathic arthritis: results from the Nordic cohort study

**DOI:** 10.1186/s13075-018-1571-6

**Published:** 2018-05-03

**Authors:** Veronika Rypdal, Ellen Dalen Arnstad, Kristiina Aalto, Lillemor Berntson, Maria Ekelund, Anders Fasth, Mia Glerup, Troels Herlin, Susan Nielsen, Suvi Peltoniemi, Marek Zak, Marite Rygg, Martin Rypdal, Ellen Nordal, For the Nordic Study Group of Pediatric Rheumatology (NoSPeR), Gudmund Marhaug, Freddy Karup Pedersen, Pekka Lahdenne, Boel Anderson-Gäre, Nils Thomas Songstad, Astri Lang, Anne Elisabeth Ross, Kjell Berntzen, Nina Moe, Mikael Damgaard, Nils Olof Jonsson, Anders Berner, Hans Ekström, Eric Ronge, Agne Lind, Lars Hammarèn, Johan Robinsson, Anna-Lena Nilsson

**Affiliations:** 1 Department of Pediatrics, University Hospital of North Norway, Tromsø, Norway; 2 Department of Clinical Medicine, UIT the Arctic University of Norway, Tromsø, Norway; 3 Department of Clinical and Molecular Medicine, NTNU - Norwegian University of Science and Technology, Trondheim, Norway; 4 Department of Pediatrics, Levanger Hospital, Nord-Trøndelag Hospital Trust, Levanger, Norway; 5 Hospital for Children and Adolescents, University of Helsinki, Helsinki, Finland; 6 Department of Women’s and Children’s Health, Uppsala University, Uppsala, Sweden; 7 Department of Pediatrics, Ryhov County Hospital, Jonkoping, Sweden; 8 Department of Pediatrics, Institute of Clinical Sciences, Sahlgrenska Academy, University of Gothenburg, Gothenburg, Sweden; 9 Department of Pediatrics, Aarhus University Hospital, Aarhus, Denmark; 10 Department of Pediatrics, Rigshospitalet Copenhagen University Hospital, Copenhagen, Denmark; 11 Department of Pediatrics, St. Olavs Hospital, Trondheim, Norway; 12 Department of Mathematics and Statistics, UIT the Arctic University of Norway, Tromsø, Norway

**Keywords:** Juvenile idiopathic arthritis, Disease activity, Prediction, Outcome research

## Abstract

**Background:**

The aim was to develop prediction rules that may guide early treatment decisions based on baseline clinical predictors of long-term unfavorable outcome in juvenile idiopathic arthritis (JIA).

**Methods:**

In the Nordic JIA cohort, we assessed baseline disease characteristics as predictors of the following outcomes 8 years after disease onset. Non-achievement of remission off medication according to the preliminary Wallace criteria, functional disability assessed by Childhood Health Assessment Questionnaire (CHAQ) and Physical Summary Score (PhS) of the Child Health Questionnaire, and articular damage assessed by the Juvenile Arthritis Damage Index-Articular (JADI-A). Multivariable models were constructed, and cross-validations were performed by repeated partitioning of the cohort into training sets for developing prediction models and validation sets to test predictive ability.

**Results:**

The total cohort constituted 423 children. Remission status was available in 410 children: 244 (59.5%) of these did not achieve remission off medication at the final study visit. Functional disability was present in 111/340 (32.7%) children assessed by CHAQ and 40/199 (20.1%) by PhS, and joint damage was found in 29/216 (13.4%). Model performance was acceptable for making predictions of long-term outcome. In validation sets, the area under the curves (AUCs) in the receiver operating characteristic (ROC) curves were 0.78 (IQR 0.72–0.82) for non-achievement of remission off medication, 0.73 (IQR 0.67–0.76) for functional disability assessed by CHAQ, 0.74 (IQR 0.65–0.80) for functional disability assessed by PhS, and 0.73 (IQR 0.63–0.76) for joint damage using JADI-A.

**Conclusion:**

The feasibility of making long-term predictions of JIA outcome based on early clinical assessment is demonstrated. The prediction models have acceptable precision and require only readily available baseline variables. Further testing in other cohorts is warranted.

**Electronic supplementary material:**

The online version of this article (10.1186/s13075-018-1571-6) contains supplementary material, which is available to authorized users.

## Background

Juvenile idiopathic arthritis (JIA) is a heterogeneous childhood disease, with chronic joint inflammation as the common feature. The JIA categories differ by the number of joints affected, and the presence of extra-articular involvement [1]. Disease course and prognosis differ between JIA categories, but there is also large variability within each category [2, 3]. Therefore, efforts have been made to discern baseline clinical prognostic factors that can predict the severity, course, and long-term outcome of the disease [4, 5].

The primary goal of JIA treatment is to achieve remission [6]. Early prediction of the disease course for the individual child can facilitate tailored treatment. There is increasing evidence for the concept of “the window of therapeutic opportunity” in JIA, where early aggressive treatment with biologic agents and/or other disease-modifying anti-rheumatic drugs (DMARDs) may modify the disease course and improve long-term prognosis [7–9]. On the other hand, it is also essential to avoid unnecessary, costly, and potentially toxic treatment in children with a favorable prognosis.

Guzman et al. have recently presented a model for prediction of severe disease course, with outcomes developed specifically for their study [10]. In a systematic literature review, Dijkhuizen and Wulffraat state the need for prospective longitudinal studies of baseline clinical predictors using standardized validated outcome measures [4]. In the Nordic JIA cohort, we studied prediction of four established and validated outcomes, and aimed to construct prediction models that may aid decision on early aggressive treatment.

## Methods

### Study population

The initial prospective longitudinal multicenter Nordic JIA cohort consisted of consecutive children with incident JIA from 12 participating centers in defined geographical areas of Denmark, Finland, Norway and Sweden. All children in these areas with newly diagnosed JIA and disease onset in the study periods between 1 January 1997 and 30 June 2000 were included. The study was designed to be as close to population-based as possible, as previously reported [11].

In the current study, 440 children met the criteria of having a baseline study visit and a final study visit 8 years after disease onset. Out of these, 17 patients with systemic JIA were excluded, because systemic JIA is considered to have autoinflammatory rather than autoimmune disease mechanisms, and the clinical characteristics of predominantly fever, rash and serositis differs from other JIA-categories [12].

The baseline study visit was planned 6 months after disease onset. At this visit, disease activity variables, complete joint count, physician’s global assessment of disease activity (physician’s GA) on a 10-cm visual analogue scale (VAS), patient’s/parent’s global assessment (GA), medication and blood tests were registered [13]. Disease onset was defined as the time of presentation of symptoms of active arthritis, and the JIA categories were determined according to the International League of Associations for Rheumatology (ILAR) criteria [14].

### Outcomes

At follow up, we evaluated 4 outcomes: (1) the main outcome was non-achievement of remission off medication, chosen as the best available validated measure of an adverse disease state over time. This included active disease, inactive disease of less than 12 months of duration, and clinical remission on medication (according to the preliminary Wallace criteria) [15, 16]. For the remainder of the paper, not in remission or non-achievement of remission refers to non-achievement of remission off medication unless otherwise specified; (2) and (3) functional disability was evaluated using the Childhood Health Assessment Questionnaire (CHAQ), and the Child Health Questionnaire Parent form (CHQ-PF50), aiming to achieve a broad evaluation of functional disability using both the JIA-specific CHAQ and the generic CHQ-PF50 instruments. CHAQ addresses functional ability in different activities of everyday life [17]. The CHAQ was completed by children of age >9 years, and otherwise by their parents, and the corresponding Health Assessment Questionnaire (HAQ) by participants > 18 years of age. From this point on in the text, CHAQ will refer to both the CHAQ and HAQ scores. The CHQ-PF50 consists of 50 items and 12 domains assessing health-related quality of life, yielding a physical summary score (PhS) and a psychological summary score (PsS) [17]. PhS ranges from 0 to 100, with a higher score indicating better functional ability; and (4) joint damage was assessed using the Juvenile Arthritis Damage Index of articular damage (JADI-A) ranging from 0 to a maximum of 72, where 36 joints, or joint groups, are scored 0 for no damage, 1 for partial damage, or 2 for severe damage [18]. All 4 outcomes were dichotomized; remission was dichotomized into clinical remission (those achieving remission without medication), and non-achievement of remission off medication (those not achieving remission or achieving remission on medication), CHAQ and JADI-A into score = 0, indicating no functional disability or no joint damage, and positive score >0, PhS into good functional ability, defined as score ≥40, and functional disability <40. This latter cutoff level is based on a reference score of 40 being one standard deviation below the mean score of healthy children in the USA [19].

### Laboratory tests

Antinuclear antibodies (ANA) and rheumatoid factor (RF) were tested at least twice with a minimum of 3 months apart. ANA was analyzed by immunofluorescence on Hep-2 cells. Tests were interpreted according to cutoff values of the local immunological laboratories. HLA-B27 was analyzed using standardized methods [20]. C-reactive protein (CRP) was measured with immunoassays, with values <10 mg/L considered normal.

### Statistics

Conventional descriptive statistics (absolute numbers, median, 1st and 3rd quartile, and percentage) were used to describe demographics and clinical characteristics. Univariate logistic regression was performed to assess baseline variables as predictors for each outcome. Variables that were significant at *p* < 0.05 in the univariate analysis were considered as candidates in a prediction model.

For each outcome, multivariable logistic regression models were constructed using a combination of predefined core variables, and additional variables selected using a forward stepwise selection method. Since the predictive ability of the models is assessed using cross-validation, the conventional limitations related to the screening of a large number of covariates in multivariable models are evaded [21]. Cross-validation controlled for overfitting of the data (internal validation), and the degree of overfitting is reflected in the performance in validation sets.

**Fig. 1:**
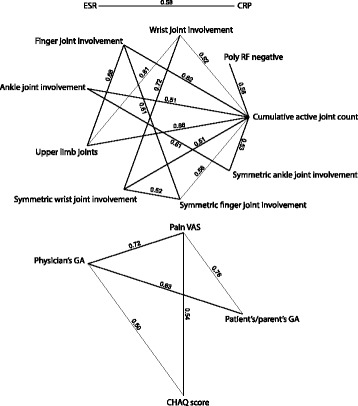
Correlations between baseline variables. Lines are drawn only between pairs of baseline variables for which the sample Spearman correlation coefficient is ≥ 0.50. Baseline variables without correlation ≥0.50 are not included in the figure. RF, rheumatoid factor; VAS, visual analogue scale; GA, global assessment; CHAQ, Childhood Health Assessment Questionnaire

Clinical characteristics included in the Wallace provisional criteria for remission were a priori included in the prediction models; the cumulative active joint count, erythrocyte sedimentation rate (ESR), CRP, physician’s GA, and morning stiffness [22]. Uveitis activity applies only to a minority of the cohort and was therefore not included. The additional baseline variables were included in a stepwise fashion if they contributed to the multivariable model with *p* < 0.05 when included. Symmetric joint involvement was not considered a candidate predictor as it correlates strongly with the specific joint involvement (Fig. 1). To ensure model simplicity the total number of variables was not allowed to exceed 10. Once the set of variables were selected, the model coefficients *β*_*i*_ for each predictor variable *x*_*i*_ were estimated using multivariable logistic regression, and the probability of unfavorable outcome was given as:

$$ P=1/\left(1+{e}^{-A}\right),\mathrm{where}\ A={\beta}_0+{\beta}_1{x}_1+\dots +{\beta}_n{x}_n. $$

For each of the four outcomes, cross-validation of the method was performed by partitioning the cohort randomly in training sets consisting of three quarters of the patients (*N* = 317) and validation sets consisting of one quarter of the patients (*N* = 106). In each realization of the random partitioning we constructed prediction models using the algorithm described above, using *only* the training set to select variables and estimate coefficients. For each of the patients in the corresponding validation set the multivariable logistic model provides a probability of the unfavorable outcome. By comparing the predicted probability of unfavorable outcome with the actual outcome at the final study visit, the receiver operating characteristic (ROC) curve was computed, and the area under the curve (AUC) was estimated. The median AUC with interquartile range (IQR) was estimated from 100 realizations of the random partitioning of the cohort. For each step in the cross-validation we omitted any patients where the outcome or the required predictor variables were not available.

Finally, in our cohort we tested the prediction model for severe disease course developed by Guzman et al. [10]. We tested Guzman’s model using the 4 outcome measures described above, i.e. not the outcomes for which their model was constructed. The analysis was performed using the software packages STATA version 14, and Wolfram Mathematica version 11.1.1.0.

### Ethical considerations

Approvals from medical research ethical committees and data protection authorities were granted according to the regulations of each participating country. Written informed consent was obtained from parents of children aged < 16 years, and from the children themselves if aged ≥ 16 years of age.

## Results

The main finding is that in the Nordic cohort, long-term outcome in JIA can be predicted, with acceptable sensitivity and specificity, using only a handful of readily available clinical variables.

### Study cohort

Characteristics of the 440 patients in the cohort have previously been published [11]. The study cohort constituted 423 children, after 17 patients with systemic JIA were excluded. The median time between disease onset and the baseline study visit was 7 (IQR 6–8) months, and between disease onset and the final study visit it was 98 (IQR 95–102) months. The median time from disease onset to diagnosis was 1.6 (IQR 0.5–3.3) months. A total of 280 patients (66.2%) were female, and the median age of disease onset in the cohort was 5.5 (IQR 2.5–9.7) years (Additional file 1: Table S1).

At the baseline study visit, 227/423 patients (53.7%) had oligoarthritis, 94/423 (22.2%) had rheumatoid factor (RF)-negative polyarthritis, and 4/423 (1.0%) had RF-positive polyarthritis (Additional file 1: Table S1). The median cumulative number of active joints within the first visit was 3 (IQR 1–6), and 381/423 patients (90.1%) had one or more affected lower limb joints at the baseline visit. Antinuclear antibodies (ANA) were present in 115/410 patients (28.1%), and HLA-B27 in 85/393 patients (21.6%) [23], presented in Additional file 1: Table S1. None of the children had started biologic agents before the baseline study visit, and early medications are shown in Additional file 2: Table S2. A total of 410/423 (96.9% of the total cohort) had baseline assessments and data on remission 8 years after disease onset. The corresponding numbers were 340/423 (80.4%) for CHAQ, 199/423 (47.0%) for PhS and 216/423 (51.1%) for JADI-A.

### Correlation between baseline variables

The clinical predictor variables were analyzed with respect to correlation. There was significantly positive, moderate to strong correlation between several variables, especially between cumulative number of active joints, the joint-specific variables, and the polyarthritis RF-negative category. Physician’s GA and the patient-reported outcomes also correlated positively with each other. The correlation structure between the predictor variables is illustrated in Fig. 1.

### Prediction of non-achievement of remission off medication

Remission status at the final study visit was available for 410 patients. There were 166 (40.5%) children in remission without medication, while 38 (9.3%) were in remission on medication, and 206 (50.2%) were not in remission: 244/410 children (59.5%) did not achieve remission off medication. The baseline predictors of not achieving remission off medication were analyzed by univariate logistic regression and are presented in Table 1.

**Table 1:** Baseline clinical characteristics as predictors of non-achievement of remission off medication in univariate logistic regression

Baseline characteristics	Total *N*	Remission off medication^a^	Not in remission^b^	OR (95% CI)	*p*
Gender female, *n* (%)	410	106 (38.8)	167 (61.2)	0.8 (0.5–1.2)	0.334
Age at disease onset, years	410	6.3 (2.5–10.0)	5.2 (2.5–9.6)	0.9 (0.9–1.0)	0.401
Time from onset to diagnosis, months	388	1.5 (0.5–2.9)	1.7 (0.5–3.6)	1.0 (1.0–1.1)	0.152
Cumulative active joint count	410	2 (1–4)	4 (2–7)	1.1 (1.1–1.2)	< 0.001
Physician’s global assessment VAS	227	0.8 (0.0–1.3)	2.0 (1.0–3.8)	3.5 (1.9–6.2)	< 0.001
Polyarticular RF-positive, *n* (%)	410	1 (25.0)	3 (75.0)	2.1 (0.2–20.0)	0.535
Polyarticular RF-negative, *n* (%)	410	25 (26.9)	68 (73.1)	2.2 (1.3–3.6)	0.003
Oligoarticular, *n* (%)	410	107 (49.1)	111 (50.9)	0.5 (0.3–0.7)	< 0.001
Psoriatic arthritis, n (%)	410	3 (50.0)	3 (50.0)	0.7 (0.1–3.4)	0.635
Enthesitis-related arthritis (ERA), *n* (%)	410	11 (32.4)	23 (67.6)	1.5 (0.7–3.1)	0.315
Undifferentiated arthritis, *n* (%)	410	19 (34.5)	36 (65.5)	1.3 (0.7–2.4)	0.336
ANA-positive, ≤ 6 years, *n* (%)^c^	397	22 (31.4)	48 (68.6)	1.6 (0.9–2.7)	0.107
Specific joint involvement, *n* (%)					
Hip joint	409	18 (32.1)	38 (67.9)	1.5 (0.8–2.8)	0.168
Ankle joint	409	57 (31.0)	127 (69.0)	2.1 (1.4–3.1)	< 0.001
Tarsal joint	409	6 (16.7)	30 (83.3)	3.8 (1.5–9.2)	0.004
Subtalar joint	409	14 (26.9)	38 (73.1)	2.0 (1.1–3.8)	0.034
Wrist joint	409	33 (30.6)	75 (69.4)	1.8 (1.1–2.9)	0.014
Finger joint	409	36 (27.7)	94 (72.3)	2.3 (1.5–3.6)	< 0.001
Neck	409	9 (26.5)	25 (73.5)	2.0 (0.9–4.4)	0.085
Upper limb joints	410	67 (32.7)	138 (67.3)	1.9 (1.3–2.9)	0.001
Lower limb joints	410	144 (39.0)	225 (61.0)	1.8 (0.9–3.5)	0.073
Symmetric involvement, *n* (%)					
Hip joints	409	5 (21.7)	18 (78.3)	2.6 (0.9–7.1)	0.067
Ankle joints	409	27 (28.4)	68 (71.6)	2.0 (1.2–3.3)	0.006
Wrist joints	409	22 (34.4)	42 (65.6)	1.4 (0.8–2.4)	0.272
Finger joints	409	13 (22.0)	46 (78.0)	2.7 (1.4–5.3)	0.002
Patient-reported outcomes					
Patient’s/parent’s global assessment VAS	250	0.5 (0.0–2.2)	1.7 (0.5–3.5)	2.2 (1.4–3.4)	0.001
CHAQ score	257	0.1 (0.0–0.6)	0.5 (0.0–1.1)	2.0 (1.3–3.0)	0.002
Pain VAS	246	0.4 (0.0–3.0)	2.3 (0.5–4.2)	1.9 (1.3–2.8)	0.002
Morning stiffness for > 15 min, *n* (%)	314	25 (22.1)	88 (77.9)	3.6 (2.1–6.0)	< 0.001
Laboratory tests					
ESR mm/h	332	11.0 (6.0–18.0)	17.0 (9.5–34.0)	1.4 (1.2–1.7)	< 0.001
CRP >10 mg/L, *n* (%)	329	12 (16.7)	60 (83.3)	3.9 (2.0–7.5)	< 0.001
ANA-positive, *n* (%)	397	37 (33.0)	75 (67.0)	1.5 (1.0–2.4)	0.075
RF-positive, *n* (%)	221	5 (50.0)	5 (50.0)	0.6 (0.2–2.0)	0.376
HLA-B27 positive, *n* (%)	382	21 (25.9)	60 (74.1)	2.1 (1.2–3.6)	0.010

Values are the median (interquartile range, IQR), or number (percentage)

*OR* odds ratio, *CI* confidence interval, *VAS* visual analogue scale, *CHAQ* Childhood Health Assessment Questionnaire, *ESR* erythrocyte sedimentation rate for an increase in 10 mm/h, *CRP* C-reactive protein, *ANA* antinuclear antibody, *RF* rheumatoid factor, *HLA-B27* human leucocyte antigen

^a^Inactive disease off medication for 12 months according to the preliminary Wallace criteria

^b^Not in remission equals non-achievement of remission off medication

^c^ANA-positive patients ≤6 years at disease onset, with oligoarticular, polyarticular RF negative, psoriatic or undifferentiated arthritis

**Fig. 2:**
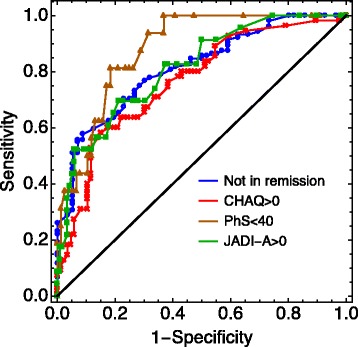
Receiver operating characteristic (ROC) curves for the four unfavorable clinical outcomes in the total cohort. Non-achievement of remission off medication; CHAQ, Childhood Health Assessment Questionnaire; PhS, Physical Summary Score; JADI-A, Juvenile Arthritis Damage Index-Articular

**Fig. 3:**
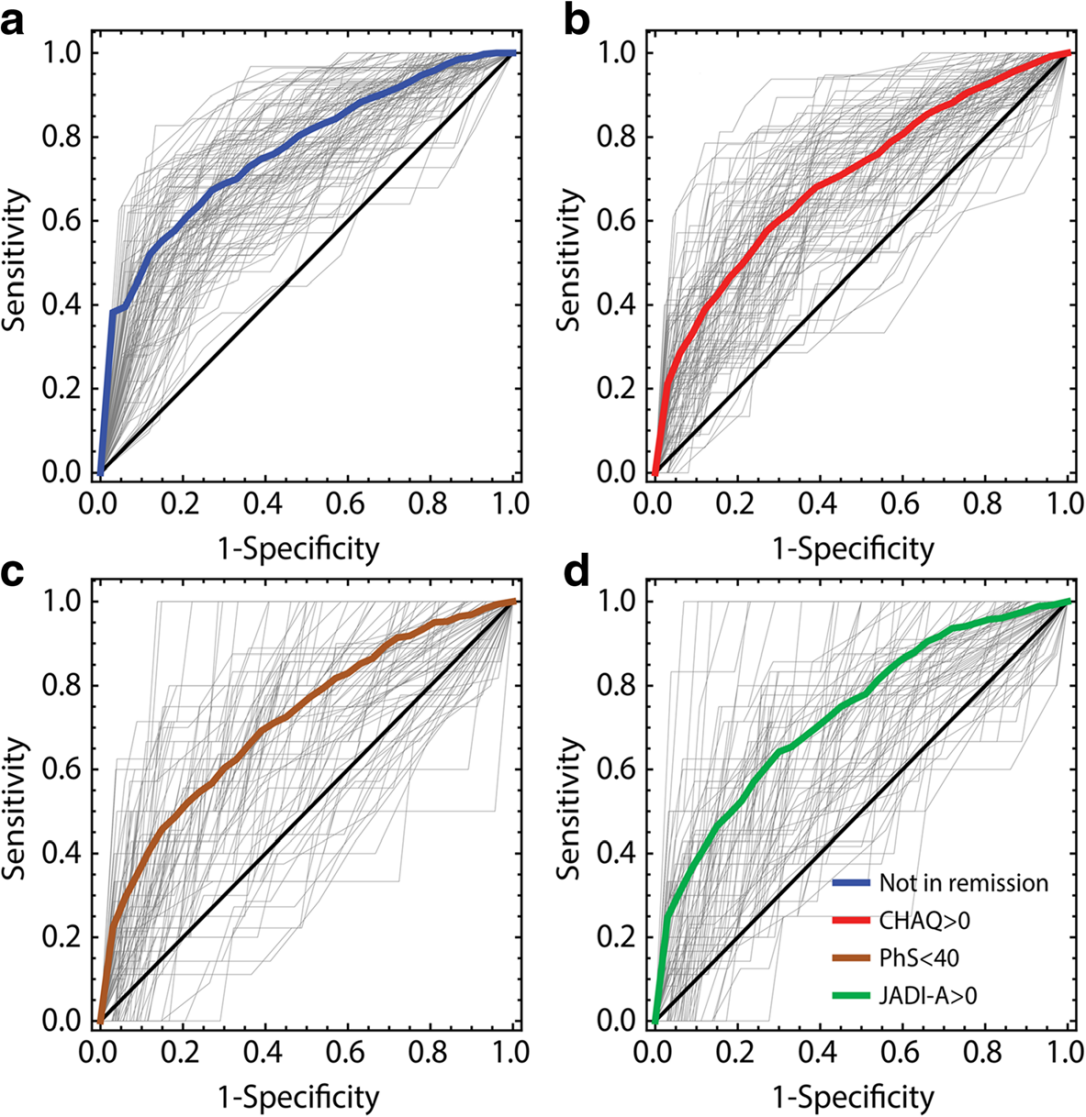
Receiver operating characteristic (ROC) curves for the four unfavorable clinical outcomes in the validation sets. The colored lines are the mean ROC curves for the 100 different realizations of the partitioning of the cohort into training sets and validation sets (thin gray curves). **a** Not in remission. **b** Childhood Health Assessment Questionnaire (CHAQ) >0. **c** Physical Summary Score (PhS) <40. **d** Juvenile Arthritis Damage Index-Articular (JADI-A) >0

The following predictor variables were included in the multivariable prediction model for non-achievement of remission: Cumulative active joint count, ESR, CRP, morning stiffness, physician’s GA, ANA, HLA-B27, and ankle joint arthritis. The first five variables were chosen a priori, and ANA, HLA-B27, and ankle joint arthritis were the variables included through the stepwise selection method (Table 2). The model has an AUC of 0.84 in the total cohort. Cross-validation yielded a median AUC = 0.78 (IQR 0.72–0.82) in the validation sets (Table 3). The corresponding ROC curves are shown in Figs. 2 and 3.

**Table 2:** Prediction of unfavorable outcome by multivariable modeling of baseline clinical characteristics

	Coef.	Std.Err
**Not in remission** ^**a**^ ***N*** ** = 156**		
	*β*_0_=-1.58	0.44
Cumulative active joint count	*β*_1_=0.04	0.05
ESR mm/h	*β*_2_=0.03	0.02
CRP >10 mg/L	*β*_3_=-0.07	0.69
Morning stiffness > 15 min	*β*_4_=1.16	0.45
Physician’s global assessment VAS	*β*_5_=0.16	0.46
ANA-positive	*β*_6_=1.25	0.50
HLA-B27-positive	*β*_7_=1.37	0.54
Ankle joint arthritis	*β*_8_=1.10	0.49
**Functional disability (CHAQ),** ***N*** ** = 141**		
	*β*_0_=-1.68	0.35
Cumulative active joint count	*β*_1_=-0.02	0.03
ESR mm/h	*β*_2_=0.01	0.01
CRP > 10 mg/L	*β*_3_=-0.20	0.63
Morning stiffness > 15 min	*β*_4_=1.03	0.42
Physician’s global assessment VAS	*β*_5_=-0.40	0.56
Finger joint arthritis	*β*_6_=1.21	0.54
Pain VAS	*β*_7_ = 0.77	0.40
**Functional disability (PhS),** ***N*** ** = 92**		
	*β*_0_=-3.40	0.75
Cumulative active joint count	*β*_1_=0.10	0.05
ESR mm/h	*β*_2_=0.01	0.02
CRP > 10 mg/L	*β*_3_=-2.06	1.28
Morning stiffness > 15 min	*β*_4_=1.68	0.80
Physician’s global assessment VAS	*β*_5_=-0.71	0.88
Pain VAS	*β*_6_=1.30	0.64
**Joint damage (JADI-A),** ***N*** ** = 141**		
	*β*_0_=-3.84	0.76
Cumulative active joint count	*β*_1_=0.02	0.04
ESR mm/h	*β*_2_=0.01	0.02
CRP > 10 mg/l	*β*_3_= -0.11	0.83
Morning stiffness > 15 min	*β*_4_=-0.59	0.61
Physician’s global assessment VAS	*β*_5_=0.28	0.52
Finger joint arthritis	*β*_6_=1.84	0.68
Older age at disease onset (years)	*β*_7_= 0.16	0.07

*Coef.* coefficients in the logistic regression, *Std.Err.* standard error in the coefficients, *VAS* visual analogue scale, *ESR* erythrocyte sedimentation rate for an increase in 10 mm/h, *CRP* C-reactive protein, *ANA* antinuclear antibody, *HLA-B27* human leucocyte antigen

^a^Not in remission equals non-achievement of remission off medication

**Table 3:** Cross-validation of the four prediction models of unfavorable long-term outcome in the Nordic JIA cohort

	Not in remission^a^	Functional disability (CHAQ)	Functional disability (PhS)	Joint damage (JADI-A)
AUC total cohort	0.84	0.79	0.90	0.84
AUC validation sets^b^	0.78 (0.72–0.82)	0.73 (0.67–0.76)	0.74 (0.65–0.80)	0.73 (0.63–0.76)

*AUC* area under the receiver operating characteristic curve, *CHAQ* Childhood Health Assessment Questionnaire, *PhS* Physical Summary Score, *JADI-A* Juvenile Arthritis Damage Index-Articular

^a^Not in remission equals non-achievement of remission off medication

^b^The AUCs in the validation sets are the median AUCs with the interquartile range of the 100 constructed models

We also developed a prediction model without the blood samples (ESR, CRP, ANA, and HLA-B27). This model yielded an AUC = 0.76 (IQR 0.72–0.80) for non-achievement of remission in the validation sets (Additional file 3: Figure S1).

### Prediction of functional disability and joint damage

The CHAQ score at the final study visit was available in 340 children, and 111 (32.7%) had a CHAQ score >0. Three of the four patients with RF-positive polyarthritis reported functional disability. For univariate logistic regression results see Additional file 4: Table S3.

The prediction model for CHAQ score >0 uses cumulative active joint count, ESR, CRP, morning stiffness, physician’s GA, finger joint arthritis, and pain VAS as variables (Table 2). The AUC of this model was 0.79 in the total cohort, and cross validation gave a median AUC of 0.73 (IQR 0.67–0.76) in the validation sets (Table 3). The ROC curve for the total cohort, and validation sets are shown in Figs. 2 and 3, respectively. The AUC for the model without blood samples was 0.72 (IQR 0.67–0.76) in the validation sets (Additional file 3: Figure S1).

Of the 199 patients with a physical summary score, 40 (20.1%) had a score <40. Results of the univariate analysis with PhS <40 as the outcome variable are shown in Additional file 5: Table S4. Variables included in the prediction model for PhS were cumulative active joint count, ESR, CRP, morning stiffness, Physician’s GA, and pain VAS (Table 2). The AUC was 0.90 in the total cohort, and cross-validation gave a median AUC = 0.74 (IQR 0.65–0.80) in the validation sets (Table 3, Figs. 2 and 3). The AUC for the model without blood samples was 0.73 (0.66–0.79) in the validation sets (Additional file 3: Figure S1).

The JADI-A was collected for 216 patients at the final study visit, and 29 patients (13.4%) had joint damage registered 8 years after disease onset. The baseline predictors of joint damage are presented in Additional file 6: Table S5. In the prediction model, older age at disease onset and finger joint arthritis were included in addition to the five previously included variables (Table 2). The AUC was 0.84 in the cohort, and the median AUC was 0.73 (IQR 0.63–0.76) in the validation sets. The results are summarized in Table 3 and Figs. 2 and 3. Without blood tests the median AUC in the validation sets was 0.73 (IQR 0.63–0.80) (Additional file 3: Figure S1).

### Other prediction models

The prediction model developed by Guzman et al. [10] was tested in our cohort by testing the ability of their model to predict the four outcomes described above. The model yielded an AUC = 0.69 for prediction of not achieving remission. For CHAQ >0, PhS <40, and JADI-A >0 the AUCs were 0.68, 0.69, and 0.71, respectively (Additional file 7: Figure S2).

## Discussion

In the Nordic JIA cohort, we have developed and evaluated prediction models for long-term unfavorable outcome with acceptable sensitivity and specificity based on variables easily available at baseline, which may guide individually tailored treatment. Prediction of long-term unfavorable outcome early in the disease course may be useful in deciding when to start aggressive treatment in JIA.

To our knowledge, this is the first study on long-term prediction of well-established disease outcomes in a prospective population-based JIA cohort. Cross-validation analysis of model performance yielded AUCs of 0.78, 0.73, 0.74, and 0.73, for non-achievement of remission, CHAQ >0, PhS <40, and JADI-A >0, respectively.

An important step in developing applicable prediction models for JIA was carried out by Guzman et al. in a Canadian JIA cohort [10]. The authors recommended that their results should be tested in other JIA cohorts. We were not able to reproduce the predictive ability of their model in the Nordic JIA cohort (Additional file 7: Figure S2). One obvious reason for the discrepancy could be that Guzman’s model is constructed to predict severe disease course, and not per se, any of the four pre-established, validated adverse outcomes that we assessed. Other reasons may be differences in the population-based approach, cohort composition, or ethnicity, or overfitting of models to the cohort.

The primary goal in the treatment of children with JIA is to achieve remission off medication, and the main implication of the current study is that prediction models may be useful in guiding decisions about treatment. Previous studies have indicated that the disease course may be modified by starting appropriate treatment early [9, 24, 25]. To reach the goal of early inactive disease, a treat-to-target strategy including shared decision-making with well-informed children and parents is currently recommended [6, 9]. Even with promising advances in using gene expression profiles and biomarkers as predictors of treatment response and flare risk [26–29], the practical value of prediction based on a handful of readily available clinical variables cannot be understated.

The main strengths of our study are the use of validated outcome measures, the simplicity of the models, and the strict cross-validations. The use of validated outcomes is called for in reports on prognosis in JIA [3, 30, 31]. Model simplicity is ensured through the model construction method, where the main variables in the preliminary Wallace criteria of remission are included in the models a priori [15, 22]. The additional variables that were included in our models have independently been associated with adverse outcomes in previous studies [4, 23, 32–36].

The model performance was assessed using cross-validations, where predictions were performed on validation sets that were completely separate from the data used to construct the models. The 100 repeated model constructions and evaluations prevent overfitting the data. Despite the strictness of the model-developing procedure, we still obtained acceptable predictive ability. The robustness and applicability of the prediction rules are emphasized by the fact that when the analyses were repeated without any blood tests, the performance was similar. An online calculator based on our models is available at the web-page http://predictions.no. An iOS app is also designed, and the test versions are available on request.

One of the limitations of our study is that for some of the patients, the baseline study visit scheduled 6 months after disease onset was not the first clinical visit. Some children had therefore already started treatment, mostly with nonsteroidal anti-inflammatory drugs (NSAIDs) or intraarticular corticosteroids, and were not treatment naïve when the predictor variables were assessed. This baseline time point, however, allowed use of the cumulative active joint count during the first 6 months of the disease, which is an important measure of early disease severity in line with the International League of Associations for Rheumatology (ILAR) criteria. A limitation is also that the primary outcome, non-achievement of remission off medication, is defined as inactive disease for more than 1 year, and this outcome does not necessarily reflect the disease course during the whole 8-year period. In addition, JADI-A is a rather crude measure of joint damage, and future predictive studies should therefore include imaging in joint damage assessment. Finally, the treatment given during the disease course may have altered the disease outcome, even though biologic medications were not generally available in the beginning of the study period in 1997. The natural history of JIA disease course without treatment is clearly impossible and unethical to study.

## Conclusion

We have developed statistical models for predicting non-achievement of remission off medication, functional disability, and joint damage in children with JIA. The models are easy to use, and may provide a valuable tool to aid early treatment decisions on the need for DMARDs including biologic agents if validation in other JIA cohorts and across ethnicities can confirm our results [37]. We encourage further testing of our models before the applicability can be generalized and recommended.

## Additional files

Additional file 1: **Table S1.** Characteristics of the 423 children in the Nordic juvenile idiopathic arthritis cohort at baseline. (PDF 134 kb)

Additional file 2: **Table S2.** Medications given before the baseline study visit. (PDF 108 kb)

Additional file 3: **Figure S1.** Receiver operating characteristic (ROC) curves for the four unfavorable clinical outcomes in the validation sets, but for models constructed without using blood samples as predictors. The colored lines are the mean ROC curves for the 100 different realizations of the partitioning of the cohort into training sets and validation sets (thin gray curves). (**a**) Not in remission. (**b**) Childhood Health Assessment Questionnaire (CHAQ) >0. (**c**) Physical Summary Score (PhS) <40. (**d**) Juvenile Arthritis Damage Index-Articular (JADI-A) >0. (PDF 435 kb)

Additional file 4: **Table S3.** Baseline clinical characteristics as predictors of functional disability (CHAQ) in univariate logistic regression. (PDF 125 kb)

Additional file 5: **Table S4.** Baseline clinical characteristics as predictors of functional disability (PhS) in univariate logistic regression. (PDF 163 kb)

Additional file 6: **Table S5.** Baseline clinical characteristics as predictors of joint damage (JADI-A) in univariate logistic regression. (PDF 161 kb)

Additional file 7: **Figure S2.** Receiver operating characteristic (ROC) curves for a test in the Nordic JIA cohort of the prediction model for severe disease course by Guzman et al. The area under the curve (AUC) values were 0.69 for non-achievement of remission off medication, 0.68 for Childhood Health Assessment Questionnaire (CHAQ) >0, 0.69 for Physical Summary Score (PhS) <40, and 0.71 for joint damage (JADI-A) >0. (PDF 287 kb)

## Abbreviations

ANA: Antinuclear antibodies
AUC: Area under the receiver operating characteristic curve
CHAQ: Childhood Health Assessment Questionnaire
CRP: C-reactive protein
DMARD: Disease-modifying anti-rheumatic drug
ESR: Erythrocyte sedimentation rate
GA: Global assessment
ILAR: International League of Associations for Rheumatology
IQR: Interquartile range
JADI-A: Juvenile Arthritis Damage Index-Articular
JIA: Juvenile idiopathic arthritis
NSAIDs: Nonsteroidal anti-inflammatory drugs
PhS: Physical Summary Score
RF: Rheumatoid factor
ROC: Receiver operating characteristics
VAS: Visual analogue scale

## References

[1] Prakken B, Albani S, Martini A (2011). Juvenile idiopathic arthritis. Lancet.

[2] Adib N, Silman A, Thomson W (2005). Outcome following onset of juvenile idiopathic inflammatory arthritis: I. frequency of different outcomes. Rheumatology (Oxford).

[3] Adib N, Silman A, Thomson W (2005). Outcome following onset of juvenile idiopathic inflammatory arthritis: II. predictors of outcome in juvenile arthritis. Rheumatology (Oxford).

[4] van Dijkhuizen EH, Wulffraat NM (2015). Early predictors of prognosis in juvenile idiopathic arthritis: a systematic literature review. Ann Rheum Dis.

[5] Flato B, Lien G, Smerdel A, Vinje O, Dale K, Johnston V, Sorskaar D, Moum T, Ploski R, Forre O (2003). Prognostic factors in juvenile rheumatoid arthritis: a case-control study revealing early predictors and outcome after 14.9 years. J Rheumatol.

[6] Consolaro A, Negro G, Lanni S, Solari N, Martini A, Ravelli A (2012). Toward a treat-to-target approach in the management of juvenile idiopathic arthritis. Clin Exp Rheumatol.

[7] Wallace CA, Giannini EH, Spalding SJ, Hashkes PJ, O'Neil KM, Zeft AS, Szer IS, Ringold S, Brunner HI, Schanberg LE (2012). Trial of early aggressive therapy in polyarticular juvenile idiopathic arthritis. Arthritis Rheum.

[8] Wallace CA, Giannini EH, Spalding SJ, Hashkes PJ, O'Neil KM, Zeft AS, Szer IS, Ringold S, Brunner HI, Schanberg LE (2014). Clinically inactive disease in a cohort of children with new-onset polyarticular juvenile idiopathic arthritis treated with early aggressive therapy: time to achievement, total duration, and predictors. J Rheumatol.

[9] Albers HM, Wessels JA, van der Straaten RJ, Brinkman DM, Suijlekom-Smit LW, Kamphuis SS, Girschick HJ, Wouters C, Schilham MW, le Cessie S (2009). Time to treatment as an important factor for the response to methotrexate in juvenile idiopathic arthritis. Arthritis Rheum.

[10] Guzman J, Henrey A, Loughin T, Berard RA, Shiff NJ, Jurencak R, Benseler SM, Tucker LB, Re A-OI (2017). Predicting which children with juvenile idiopathic arthritis will have a severe disease course: results from the ReACCh-Out Cohort. J Rheumatol.

[11] Nordal E, Zak M, Aalto K, Berntson L, Fasth A, Herlin T, Lahdenne P, Nielsen S, Straume B, Rygg M (2011). Ongoing disease activity and changing categories in a long-term nordic cohort study of juvenile idiopathic arthritis. Arthritis Rheum.

[12] Vastert SJ, Kuis W, Grom AA (2009). Systemic JIA: new developments in the understanding of the pathophysiology and therapy. Best Pract Res Clin Rheumatol.

[13] Giannini EH, Ruperto N, Ravelli A, Lovell DJ, Felson DT, Martini A (1997). Preliminary definition of improvement in juvenile arthritis. Arthritis Rheum.

[14] Petty RE, Southwood TR, Manners P, Baum J, Glass DN, Goldenberg J, He X, Maldonado-Cocco J, Orozco-Alcala J, Prieur AM (2004). International League of Associations for Rheumatology classification of juvenile idiopathic arthritis: second revision, Edmonton, 2001. J Rheumatol.

[15] Wallace CA, Ravelli A, Huang B, Giannini EH (2006). Preliminary validation of clinical remission criteria using the OMERACT filter for select categories of juvenile idiopathic arthritis. J Rheumatol.

[16] Wallace CA, Ruperto N, Giannini E, Childhood A, Rheumatology Research A, Pediatric Rheumatology International Trials O, Pediatric Rheumatology Collaborative Study G (2004). Preliminary criteria for clinical remission for select categories of juvenile idiopathic arthritis. J Rheumatol.

[17] Ruperto N, Ravelli A, Pistorio A, Malattia C, Cavuto S, Gado-West L, Tortorelli A, Landgraf JM, Singh G, Martini A (2001). Cross-cultural adaptation and psychometric evaluation of the Childhood Health Assessment Questionnaire (CHAQ) and the Child Health Questionnaire (CHQ) in 32 countries. Review of the general methodology. Clin Exp Rheumatol.

[18] Viola S, Felici E, Magni-Manzoni S, Pistorio A, Buoncompagni A, Ruperto N, Rossi F, Bartoli M, Martini A, Ravelli A (2005). Development and validation of a clinical index for assessment of long-term damage in juvenile idiopathic arthritis. Arthritis Rheum.

[19] Landgraf JMAL, Ware JE (1996). The CHQ user’s manual.

[20] Hulstaert FAJ, Hannet I, Lancaster P, Buchner L, Kunz J (1994). An optimized method for routine HLA-B27 screening using flow cytometry. Cytometry.

[21] Lydersen S. Statistical review. Frequently given comments. Ann Rheum Dis. 2015;74(2):323–5. 10.1136/annrheumdis-2014-206186. 10.1136/annrheumdis-2014-206186 25261576

[22] Wallace CA, Giannini EH, Huang B, Itert L, Ruperto N, Childhood Arthritis Rheumatology Research A, Pediatric Rheumatology Collaborative Study G, Paediatric Rheumatology International Trials O (2011). American College of Rheumatology provisional criteria for defining clinical inactive disease in select categories of juvenile idiopathic arthritis. Arthritis Care Res (Hoboken).

[23] Berntson L, Nordal E, Aalto K, Peltoniemi S, Herlin T, Zak M, Nielsen S, Rygg M, Nordic Study Group of Paediatric R (2013). HLA-B27 predicts a more chronic disease course in an 8-year followup cohort of patients with juvenile idiopathic arthritis. J Rheumatol.

[24] Bartoli M, Taro M, Magni-Manzoni S, Pistorio A, Traverso F, Viola S, Magnani A, Gasparini C, Martini A, Ravelli A (2008). The magnitude of early response to methotrexate therapy predicts long-term outcome of patients with juvenile idiopathic arthritis. Ann Rheum Dis.

[25] Tynjala P, Vahasalo P, Tarkiainen M, Kroger L, Aalto K, Malin M, Putto-Laurila A, Honkanen V, Lahdenne P (2011). Aggressive combination drug therapy in very early polyarticular juvenile idiopathic arthritis (ACUTE-JIA): a multicentre randomised open-label clinical trial. Ann Rheum Dis.

[26] Gerss J, Roth J, Holzinger D, Ruperto N, Wittkowski H, Frosch M, Wulffraat N, Wedderburn L, Stanevicha V, Mihaylova D (2012). Phagocyte-specific S100 proteins and high-sensitivity C reactive protein as biomarkers for a risk-adapted treatment to maintain remission in juvenile idiopathic arthritis: a comparative study. Ann Rheum Dis.

[27] SA JK, Frank MB, Chen Y, Wallace CA, Jarvis JN (2014). Whole blood gene expression profiling predicts therapeutic response at six months in patients with polyarticular juvenile idiopathic arthritis. Arthritis Rheumatol.

[28] Fall N, Barnes M, Thornton S, Luyrink L, Olson J, Ilowite NT, Gottlieb BS, Griffin T, Sherry DD, Thompson S (2007). Gene expression profiling of peripheral blood from patients with untreated new-onset systemic juvenile idiopathic arthritis reveals molecular heterogeneity that may predict macrophage activation syndrome. Arthritis Rheum.

[29] Anink J, Van Suijlekom-Smit LW, Otten MH, Prince FH, van Rossum MA, Dolman KM, Hoppenreijs EP, ten Cate R, Ursu S, Wedderburn LR (2015). MRP8/14 serum levels as a predictor of response to starting and stopping anti-TNF treatment in juvenile idiopathic arthritis. Arthritis Res Ther.

[30] Ravelli A, Martini A (2003). Early predictors of outcome in juvenile idiopathic arthritis. Clin Exp Rheumatol.

[31] Wallace CA, Huang B, Bandeira M, Ravelli A, Giannini EH (2005). Patterns of clinical remission in select categories of juvenile idiopathic arthritis. Arthritis Rheum.

[32] Ravelli A, Varnier GC, Oliveira S, Castell E, Arguedas O, Magnani A, Pistorio A, Ruperto N, Magni-Manzoni S, Galasso R (2011). Antinuclear antibody-positive patients should be grouped as a separate category in the classification of juvenile idiopathic arthritis. Arthritis Rheum.

[33] Alberdi-Saugstrup M, Zak M, Nielsen S, Herlin T, Nordal E, Berntson L, Fasth A, Rygg M, Klaus M, Nordic Study Group of Pediatric R (2017). High-sensitive CRP as a predictive marker of long-term outcome in juvenile idiopathic arthritis. Rheumatol Int.

[34] Esbjornsson AC, Aalto K, Brostrom EW, Fasth A, Herlin T, Nielsen S, Nordal E, Peltoniemi S, Rygg M, Zak M (2015). Ankle arthritis predicts polyarticular disease course and unfavourable outcome in children with juvenile idiopathic arthritis. Clin Exp Rheumatol.

[35] Oberle EJ, Harris JG, Verbsky JW (2014). Polyarticular juvenile idiopathic arthritis - epidemiology and management approaches. Clin Epidemiol.

[36] Beukelman T, Patkar NM, Saag KG, Tolleson-Rinehart S, Cron RQ, Dewitt EM, Ilowite NT, Kimura Y, Laxer RM, Lovell DJ (2011). American College of Rheumatology recommendations for the treatment of juvenile idiopathic arthritis: initiation and safety monitoring of therapeutic agents for the treatment of arthritis and systemic features. Arthritis Care Res (Hoboken).

[37] Consolaro A, Ruperto N, Filocamo G, Lanni S, Bracciolini G, Garrone M, Scala S, Villa L, Silvestri G, Tani D (2012). Seeking insights into the epidemiology, treatment and outcome of childhood arthritis through a multinational collaborative effort: introduction of the EPOCA study. Pediatr Rheumatol Online J.

