# Supplementary material for: Predicting unfavorable long-term outcome in juvenile idiopathic arthritis: results from the Nordic cohort study
Source: Arthritis Res Ther. 2018 May 3;20:91. doi: 10.1186/s13075-018-1571-6 (PMC5934822; doi:10.1186/s13075-018-1571-6)
Supplement: Supplementary file 1 — Table S1. Characteristics of the 423 children in the Nordic juvenile idiopathic arthritis cohort at baseline. (PDF 134 kb) [file 13075_2018_1571_MOESM1_ESM.pdf]

Additional file 1

Table S1 Characteristics of the 423 children in the Nordic juvenile idiopathic arthritis cohort at baseline

**Baseline characteristics**	**Total, *N***	**Median (IQR), or n (%)** GG
Gender female, n (%)	423	280 (66.2)
Age at disease onset, years	423	5.5 (2.5-9.7)
First study visit, months	423	7 (6-8)
Last study visit, months	423	98 (95-102)
Time from onset to diagnosis, months	399	1.6 (0.5-3.3)
Cumulative active joint count	423	3 (1-6)
Physician's global assessment VAS	234	1.1 (0.5-3.0)
**Specific joint involvement, n (%)**		
Hip joint	422	58 (13.7)
Ankle joint	422	188 (44.6)
Tarsal joint	422	36 (8.5)
Subtalar joint	422	54 (12.8)
Wrist joint	422	111 (26.3)
Finger joint	422	131 (31.0)
Neck	422	34 (8.1)
Upper limb joints	423	210 (49.7)
Lower limb joints	423	381 (90.1)
**Symmetric joint involvement, n (%)**		
Hip joints	422	23 (5.5)
Ankle joints	422	97 (23.0)
Wrist joints	422	66 (15.6)
Finger joints	422	60 (14.2)
**JIA category, n (%)**		
Oligoarticular	423	227 (53.7)
Polyarticular RF negative	423	94 (22.2)
Polyarticular RF positive	423	4 (1.0)
Psoriatic arthritis	423	6 (1.4)
Enthesitis-related arthritis (ERA)	423	34 (8.0)
Undifferentiated arthritis	423	58 (13.7)
ANA positive ≤6 years[a]	410	72 (17.6)
**Patient-reported outcomes**		
Patient's/parent's global assessment VAS	257	1.0 (0.2-3.0)
CHAQ score	264	0.4 (0.0-1.0)
Pain VAS	253	1.1 (0.1-3.8)
Morning stiffness >15 minutes, n (%)	324	113 (34.9)
**Lab tests**		
ESR mm/hour	342	14.0 (8.0-28.0)
CRP >10 mg/liter, n (%)	338	73 (21.6)
ANA positive, n (%)	410	115 (28.1)
RF positive, n (%)	228	10 (4.4)
HLA-B27 positive, n (%)	393	85 (21.6)

Values are the median (Interquartile range, IQR), or n (%). [a]ANA-positive patients ≤6 years at disease onset, with oligoarticular, polyarticular RF negative, psoriatic arthritis or undifferentiated arthritis. VAS, visual analogue scale; CHAQ, Childhood Health Assessment Questionnaire; ESR, erythrocyte sedimentation rate for an increase in 10mm/hours; CRP, C-reactive protein; ANA, antinuclear antibody; RF, rheumatoid factor; HLA-B27, human leucocyte antigen.
