# Supplementary material for: Predicting unfavorable long-term outcome in juvenile idiopathic arthritis: results from the Nordic cohort study
Source: Arthritis Res Ther. 2018 May 3;20:91. doi: 10.1186/s13075-018-1571-6 (PMC5934822; doi:10.1186/s13075-018-1571-6)
Supplement: Supplementary file 2 — Table S2. Medications given before the baseline study visit. (PDF 108 kb) [file 13075_2018_1571_MOESM2_ESM.pdf]

Additional file 2

Table S2 Medications given before the baseline study visit

**Medication**	**Total *N***	**N (%)**
NSAIDs	417	357 (85.6)
Intrarticular steroid injections	412	241 (58.0)
Steroids systemic	417	38 (9.1)
Methotrexate	402	55 (13.7)
Other DMARDs[a]	405	37 (9.1)
Systemic treatment[b]	398	92 (23.1)
Biologic treatment	422	0

[a]Other DMARDs; salazopyrin, hydroxychloroquine, gold, azatioprin, cyclosporin, cyclophosphamide

[b]Systemic treatment; DMARDs total and steroids
