# Supplementary material for: Predicting unfavorable long-term outcome in juvenile idiopathic arthritis: results from the Nordic cohort study
Source: Arthritis Res Ther. 2018 May 3;20:91. doi: 10.1186/s13075-018-1571-6 (PMC5934822; doi:10.1186/s13075-018-1571-6)
Supplement: Supplementary file 3 — Figure S1. Receiver operating characteristic (ROC) curves for the four unfavorable clinical outcomes in the validation sets, but for models constructed without using blood samples as predictors. The colored lines are the mean ROC curves for the 100 different realizations of the partitioning of the cohort into training sets and validation sets (thin gray curves). (a) Not in remission. (b) Childhood Health Assessment Questionnaire (CHAQ) >0. (c) Physical Summary Score (PhS) <40. (d) Juvenile Arthritis Damage Index-Articular (JADI-A) >0. (PDF 435 kb) [file 13075_2018_1571_MOESM3_ESM.pdf]

Additional file 3

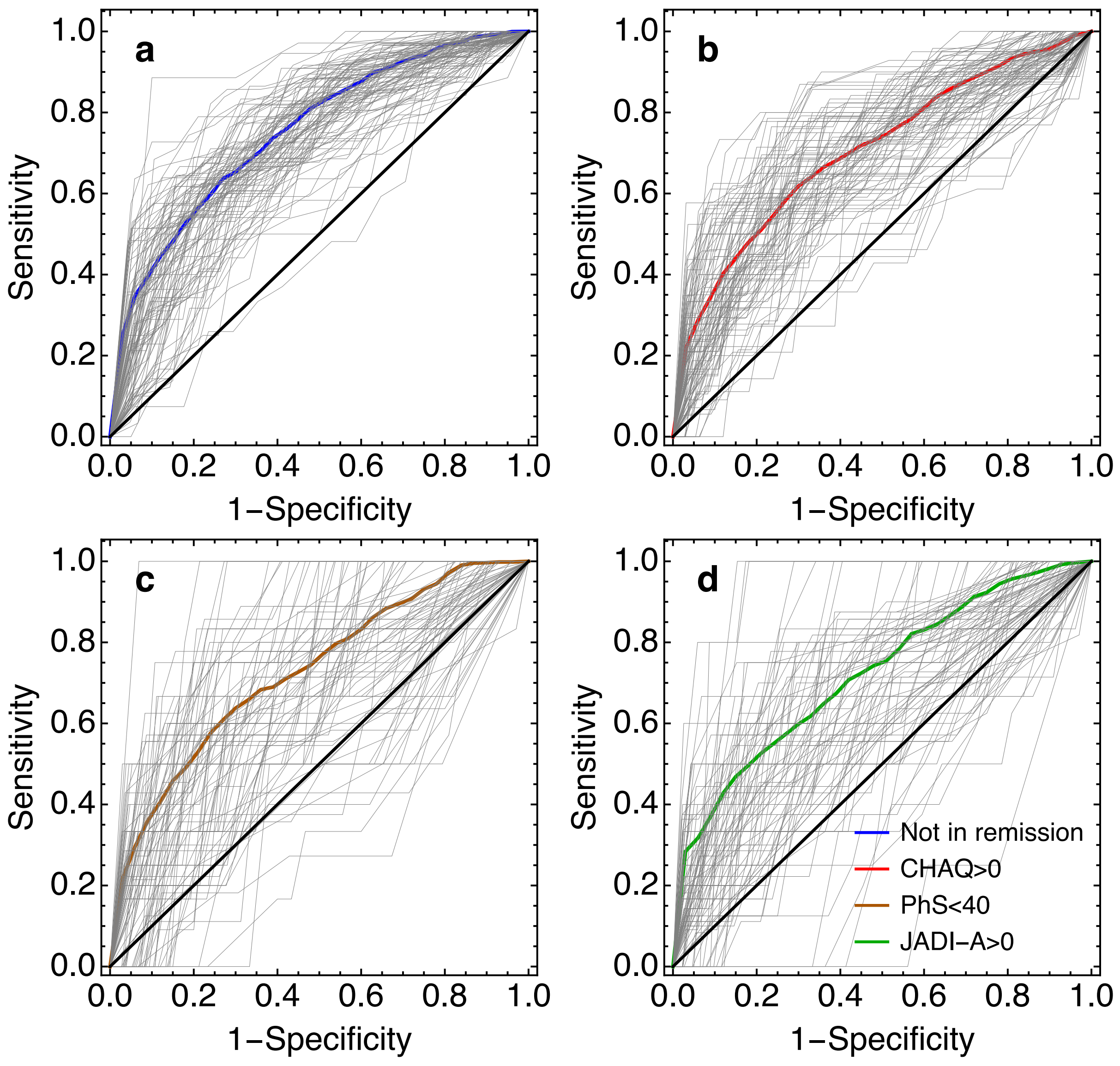

Figure S1 Receiver operating characteristics (ROC) curves for the four unfavorable clinical outcomes in the validation sets, but for model constructed without using blood samples as predictors. The colored lines are the mean ROC curves for the 100 different realizations of the partitioning of the cohort into training sets and validation sets (thin gray curves). (a): Not in remission. (b): CHAQ>0, Childhood Health Assessment Questionnaire. (c): PhS<40, Physical Summary Score. (d): JADI-A>0, Juvenile Arthritis Damage Index-Articular.
