# Supplementary material for: Predicting unfavorable long-term outcome in juvenile idiopathic arthritis: results from the Nordic cohort study
Source: Arthritis Res Ther. 2018 May 3;20:91. doi: 10.1186/s13075-018-1571-6 (PMC5934822; doi:10.1186/s13075-018-1571-6)
Supplement: Supplementary file 4 — Table S3. Baseline clinical characteristics as predictors of functional disability (CHAQ) in univariate logistic regression. (PDF 125 kb) [file 13075_2018_1571_MOESM4_ESM.pdf]

Additional file 4

Table S3 Baseline clinical characteristics as predictors of functional disability (CHAQ) in univariate logistic regression

**Baseline characteristics**	**Total *N***	**CHAQ =0**	**CHAQ>0**	**OR (95 % CI)**	***p***
Gender female, n (%)	340	142 (62.8)	84 (37.2)	0.5 (0.3-0.9)	0.013
Age at disease onset, years	340	5.2 (2.5-9.4)	5.3 (2.3-9.7)	1.0 (1.0-1.1)	0.812
Time from onset to diagnosis, months	325	1.3 (0.5-2.8)	1.9 (0.5-4.0)	1.0 (1.0-1.1)	0.106
Cumulative active joint count	340	3 (1-6)	5 (2-9)	1.1 (1.0-1.1)	0.001
Physician's global assessment VAS	199	1.0 (0.2-2.1)	2.0 (0.7-4.2)	2.6 (1.5-4.2)	<0.001
Polyarticular RF positive, n (%)	340	1 (25.0)	3 (75.0)	6.3 (0.7-61.6)	0.112
Polyarticular RF negative, n (%)	340	48 (57.8)	35 (42.2)	1.7 (1.1-2.9)	0.034
Oligoarticular, n (%)	340	128 (72.7)	48(27.3)	0.6 (0.4-0.9)	0.029
Psoriatic arthritis, n (%)	340	4 (66.7)	2 (33.3)	1.0 (0.2-5.7)	0.971
Enthesitis-related arthritis (ERA), n (%)	340	19 (76.0)	6 (24.0)	0.6 (0.2-1.6)	0.342
Undifferentiated arthritis, n (%)	340	29 (63.0)	17 (37.0)	1.2 (0.7-2.4)	0.503
ANA positive, ≤6 years, n (%)[a]	340	36 (61.0)	23 (39.0)	1.4 (0.8-2.5)	0.237
**Specific joint involvement, n (%)**					
Hip joint	339	34 (70.8)	14 (29.2)	0.8 (0.4-1.6)	0.601
Ankle joint	339	93 (61.2)	59 (38.8)	1.7 (1.1-2.7)	0.025
Tarsal joint	339	26 (78.8)	7 (21.2)	0.5 (0.2-1.3)	0.152
Subtalar joint	339	36 (76.6)	11 (23.4)	0.6 (0.3-1.2)	0.157
Wrist joint	339	46 (53.5)	40 (46.5)	2.3 (1.4-3.8)	0.001
Finger joint	339	54 (50.0)	54 (50.0)	3.1 (1.9-5.1)	<0.001
Neck	339	15 (53.6)	13 (46.4)	1.9 (0.9-4.2)	0.104
Upper limb joints	340	97 (56.7)	74 (43.3)	2.7 (1.7-4.4)	<0.001
Lower limb joints	340	202 (66.2)	103 (33.8)	1.7 (0.8-3.9)	0.197
**Symmetric involvement, n (%)**					
Hip joints	339	13 (68.4)	6 (31.6)	1.0 (0.4-2.6)	0.934
Ankle joints	339	47 (57.3)	35 (42.7)	1.8 (1.1-3.0)	0.024
Wrist joints	339	25 (48.1)	27 (51.9)	2.7 (1.5-4.8)	0.001
Finger joints	339	18 (36.7)	31 (63.3)	4.6 (2.4-8.7)	<0.001
**Patient-reported outcomes**					
Patient's/parent's global assessment VAS	223	0.8 (0.0-2.2)	2.0 (1.0-3.4)	2.2 (1.4-3.5)	<0.001
CHAQ score	228	0.1 (0.0-0.9)	0.6 (0.0-1.4)	2.0 (1.3-3.0)	0.001
Pain VAS	220	0.5 (0.0-2.5)	2.7 (1.0-5.0)	3.0 (1.9-4.8)	<0.001
Morning stiffness >15 minutes, n (%)	265	38 (40.4)	56 (59.6)	5.2 (3.0-8.9)	<0.001
**Lab tests**					
ESR mm/hour	275	14.0 (8.0-25.0)	14.5 (8.0-39.0)	1.1 (1.0-1.2)	0.142
CRP >10 mg/liter, n (%)	275	42 (64.6)	23 (35.4)	1.2 (0.7-2.1)	0.552
ANA positive, n (%)	333	57 (59.4)	39 (40.6)	1.7 (1.0-2.7)	0.043
RF positive, n (%)	184	5 (62.5)	3 (37.5)	1.0 (0.2-4.2)	0.974
HLA-B27 positive, n (%)	323	45 (65.2)	24 (34.8)	1.1 (0.6-1.9)	0.789

Values are the median (Interquartile range, IQR), or n (%). [a]ANA-positive patients ≤6 years at disease onset, with oligoarticular, polyarticular RF negative, psoriatic or undifferentiated arthritis. CHAQ, Childhood Health Assessment Questionnaire; OR, Odds ratio; CI, Confidence interval; VAS, visual analogue scale; ESR, erythrocyte sedimentation rate for an increase in 10mm/hours; CRP, C-reactive protein; ANA, antinuclear antibody; RF, rheumatoid factor; HLA-B27, human leucocyte antigen.
