# Supplementary material for: Predicting unfavorable long-term outcome in juvenile idiopathic arthritis: results from the Nordic cohort study
Source: Arthritis Res Ther. 2018 May 3;20:91. doi: 10.1186/s13075-018-1571-6 (PMC5934822; doi:10.1186/s13075-018-1571-6)
Supplement: Supplementary file 5 — Table S4. Baseline clinical characteristics as predictors of functional disability (PhS) in univariate logistic regression. (PDF 163 kb) [file 13075_2018_1571_MOESM5_ESM.pdf]

Additional file 5

Table S4 Baseline clinical characteristics as predictors of functional disability (PhS) in univariate logistic regression

**Baseline characteristics**	**Total *N***	**PhS ≥ 40**	**PhS<40**	**OR (95 % CI)**	***p***
Gender female, n (%)	199	97 (77.0)	29 (23.0)	0.6 (0.3-1.3)	0.181
Age at disease onset, years	199	3.9 (1.9-6.8)	3.0 (2.2-6.5)	1.0 (0.8-1.1)	0.603
Time from onset to diagnosis, months	188	1.0 (0.4-2.4)	2.0 (0.8-3.6)	1.1 (1.0-1.1)	0.131
Cumulative active joint count	199	3 (1-6)	5 (2-8)	1.1 (1.0-1.1)	0.035
Physician's global assessment VAS	129	1.1 (0.5-2.8)	1.1 (0.7-4.5)	1.6 (0.8-3.1)	0.149
Polyarticular RF positive, n (%)	199	1 (100)	0	NA	NA
Polyarticular RF negative, n (%)	199	40 (72.7)	15 (27.3)	1.8 (0.9-3.7)	0.122
Oligoarticular, n (%)	199	92 (85.2)	16 (14.8)	0.5 (0.2-1.0)	0.045
Psoriatic arthritis, n (%)	199	4 (100)	0	NA	NA
Enthesitis-related arthritis (ERA), n (%)	199	7 (87.5)	1 (12.5)	0.6 (0.1-4.7)	0.589
Undifferentiated arthritis, n (%)	199	15 (65.2)	8 (34.8)	2.4 (0.9-6.1)	0.068
ANA positive, ≤6 years, n (%)[a]	194	36 (75.0)	12 (25.0)	1.4 (0.6-3.0)	0.388
**Specific joint involvement, n (%)**					
Hip joint	199	19 (79.2)	5 (20.8)	1.1 (0.4-3.0)	0.924
Ankle joint	199	77 (76.2)	24 (23.8)	1.6 (0.8-3.2)	0.193
Tarsal joint	199	15 (75.0)	5 (25.0)	1.4 (0.5-4.0)	0.566
Subtalar joint	199	20 (76.9)	6 (23.1)	1.2 (0.5-3.3)	0.685
Wrist joint	199	38 (71.7)	15 (28.3)	1.9 (0.9-4.0)	0.085
Finger joint	199	41 (67.2)	20 (32.8)	2.9 (1.4-5.9)	0.004
Neck	199	9 (56.3)	7 (43.7)	3.5 (1.2-10.2)	0.019
Upper limb joints	199	71 (74.0)	25 (26.0)	2.1 (1.0-4.2)	0.046
Lower limb joints	199	149 (79.3)	39 (20.7)	2.6 (0.3-21.1)	0.366
**Symmetric involvement, n (%)**					
Hip joints	199	7 (70.0)	3 (30.0)	1.8 (0.4-7.1)	0.428
Ankle joints	199	38 (70.4)	16 (29.6)	2.1 (1.0-4.4)	0.043
Wrist joints	199	22 (71.0)	9 (29.0)	1.8 (0.8-4.3)	0.181
Finger joints	199	15 (62.5)	9 (37.5)	2.8 (1.1-6.9)	0.028
**Patient-reported outcomes**					
Patient's/parent's global assessment VAS	141	1.0 (0.0-2.7)	2.0 (0.7-5.0)	2.0 (1.1-3.7)	0.028
CHAQ score	144	0.4 (0.0-0.9)	0.4 (0.0-1.1)	1.3 (0.7-2.4)	0.390
Pain VAS	138	1.0 (0.0-2.6)	4.0 (0.9-5.0)	3.0 (1.5-5.9)	0.001
Morning stiffness >15 minutes, n (%)	166	43 (67.2)	21 (32.8)	3.1 (1.4-6.6)	0.004
**Lab tests**					
ESR mm/hour	164	14.0 (9.0-25.0)	18.0 (12.0-43.0)	1.1(1.0-1.3)	0.167
CRP >10 mg/liter, n (%)	167	29 (74.4)	10 (25.6)	1.5 (0.6-3.5)	0.351
ANA positive, n (%)	194	53 (77.9)	15 (22.1)	1.1 (0.6-2.4)	0.716
RF positive, n (%)	116	3 (100)	0	NA	NA
HLA-B27 positive, n (%)	196	27 (77.1)	8 (22.9)	1.2 (0.5-2.9)	0.692

Values are the median (Interquartile range, IQR), or n (%). [a]ANA-positive patients ≤6 years at disease onset, with oligoarticular, polyarticular RF negative, psoriatic or undifferentiated arthritis. PhS, Physical summary score; OR, Odds ratio; CI, Confidence interval; VAS, visual analogue scale; CHAQ, Childhood Health Assessment Questionnaire; ESR, erythrocyte sedimentation rate for an increase in 10mm/hours; CRP, C-reactive protein; ANA, antinuclear antibody; RF, rheumatoid factor; HLA-B27, human leucocyte antigen. NA, not applicable.
