# Supplementary material for: Predicting unfavorable long-term outcome in juvenile idiopathic arthritis: results from the Nordic cohort study
Source: Arthritis Res Ther. 2018 May 3;20:91. doi: 10.1186/s13075-018-1571-6 (PMC5934822; doi:10.1186/s13075-018-1571-6)
Supplement: Supplementary file 6 — Table S5. Baseline clinical characteristics as predictors of joint damage (JADI-A) in univariate logistic regression. (PDF 161 kb) [file 13075_2018_1571_MOESM6_ESM.pdf]

Additional file 6

Table S5 Baseline clinical characteristics as predictors of joint damage (JADI-A) in univariate logistic regression

**Baseline characteristics**	**Total *N***	**JADI-A=0**	**JADI-A>0**	**OR (95 % CI)**	***p***
Gender female, n (%)	216	126 (85.7)	21 (14.3)	0.8 (0.3-1.9)	0.589
Age at disease onset, years	216	5.1 (2.4-9.7)	8.3 (5.9-10.9)	1.1 (1.0-1.2)	0.032
Time from onset to diagnosis, months	205	1.5 (0.5-3.1)	2.2 (0.7-5.7)	1.1 (1.0-1.1)	0.093
Cumulative active joint count	216	3 (1-7)	6 (3-14)	1.1(1.0-1.2)	<0.001
Physician's global assessment VAS	193	1.0 (0.5-2.8)	2.5 (0.9-4.6)	2.0 (1.1-3.7)	0.022
Polyarticular RF positive, n (%)	216	0	2 (100)	NA	NA
Polyarticular RF negative, n (%)	216	42 (77.8)	12 (22.2)	2.4 (1.1-5.5)	0.032
Oligoarticular, n (%)	216	101 (91.0)	10 (9.0)	0.4 (0.2-1.0)	0.054
Psoriatic arthritis, n (%)	216	1 (100)	0	NA	NA
Enthesitis-related arthritis (ERA), n (%)	216	17 (89.5)	2 (10.5)	0.7 (0.2-3.4)	0.699
Undifferentiated arthritis, n (%)	216	26 (89.7)	3 (10.3)	0.7 (0.2-2.5)	0.602
ANA positive, ≤6 years, n (%)[a]	216	42 (95.5)	2 (4.5)	0.3 (0.1-1.1)	0.070
**Specific joint involvement, n (%)**					
Hip joint	215	26 (83.9)	5 (16.1)	1.3 (0.4-3.7)	0.642
Ankle joint	215	87 (86.1)	14 (13.9)	1.1 (0.5-2.3)	0.880
Tarsal joint	215	9 (75.0)	3 (25)	2.3 (0.6-8.9)	0.241
Subtalar joint	215	8 (80.0)	2 (20.0)	1.6 (0.3-8.2)	0.541
Wrist joint	215	49 (79.0)	13 (21.0)	2.3 (1.0-5.1)	0.045
Finger joint	215	52 (72.2)	20 (27.8)	5.7 (2.4-13.4)	<0.001
Neck	215	15 (62.5)	9 (37.5)	5.1 (2.0-13.2)	0.001
Upper limb joints	216	93 (80.2)	23 (19.8)	3.9 (1.5-9.9)	0.005
Lower limb joints	216	174 (86.1)	28 (13.9)	2.1 (0.3-16.6)	0.485
**Symmetric involvement, n (%)**					
Hip joints	215	12 (80.0)	3 (20.0)	1.7 (0.4-6.3)	0.448
Ankle joints	215	54 (83.1)	11 (16.9)	1.5 (0.7-3.4)	0.334
Wrist joints	215	31 (75.6)	10 (24.4)	2.6 (1.1-6.2)	0.027
Finger joints	215	24 (64.9)	13 (35.1)	5.5 (2.3-12.8)	<0.001
**Patient-reported outcomes**					
Patient's/parent's global assessment VAS	195	1.0 (0.0-3.1)	1.8 (0.9-3.3)	1.4 (0.8-2.5)	0.209
CHAQ score	199	0.3 (0.0-0.9)	0.6 (0.0-1.4)	1.9 (1.0-3.4)	0.037
Pain VAS	192	1.0 (0.0-3.8)	1.5 (0.3-4.3)	1.3 (0.7-2.3)	0.422
Morning stiffness >15 minutes, n (%)	208	74 (83.0)	15 (17.0)	1.5 (0.7-3.3)	0.297
**Lab tests**					
ESR mm/hour	167	13.0 (7.0-22.0)	15.0 (8.5-26.5)	1.1(0.9-1.3)	0.458
CRP >10 mg/liter, n (%)	169	28 (77.8)	8 (22.2)	1.7 (0.7-4.3)	0.253
ANA positive, n (%)	209	58 (87.9)	8 (12.1)	0.8 (0.4-2.0)	0.713
RF positive, n (%)	159	4 (66.7)	2 (33.3)	2.8 (0.5-16.3)	0.246
HLA-B27 positive, n (%)	214	45 (86.5)	7 (13.5)	1.0 (0.4-2.6)	0.926

Values are the median (Interquartile range, IQR), or n (%). [a]ANA-positive patients ≤6 years at disease onset, with oligoarticular, polyarticular RF negative, psoriatic or undifferentiated arthritis. JADI-A, Juvenile Arthritis Damage Index-Articular; OR, Odds ratio; CI, Confidence interval; VAS, visual analogue scale; CHAQ, Childhood Health Assessment Questionnaire; ESR, erythrocyte sedimentation rate for an increase in 10mm/hours; CRP, C-reactive protein; ANA, antinuclear antibody; RF, rheumatoid factor; HLA-B27, human leucocyte antigen. NA, not applicable.
