# Supplementary material for: Predicting unfavorable long-term outcome in juvenile idiopathic arthritis: results from the Nordic cohort study
Source: Arthritis Res Ther. 2018 May 3;20:91. doi: 10.1186/s13075-018-1571-6 (PMC5934822; doi:10.1186/s13075-018-1571-6)
Supplement: Supplementary file 7 — Figure S2. Receiver operating characteristic (ROC) curves for a test in the Nordic JIA cohort of the prediction model for severe disease course by Guzman et al. The area under the curve (AUC) values were 0.69 for non-achievement of remission off medication, 0.68 for Childhood Health Assessment Questionnaire (CHAQ) >0, 0.69 for Physical Summary Score (PhS) <40, and 0.71 for joint damage (JADI-A) >0. (PDF 287 kb) [file 13075_2018_1571_MOESM7_ESM.pdf]

Additional file 7

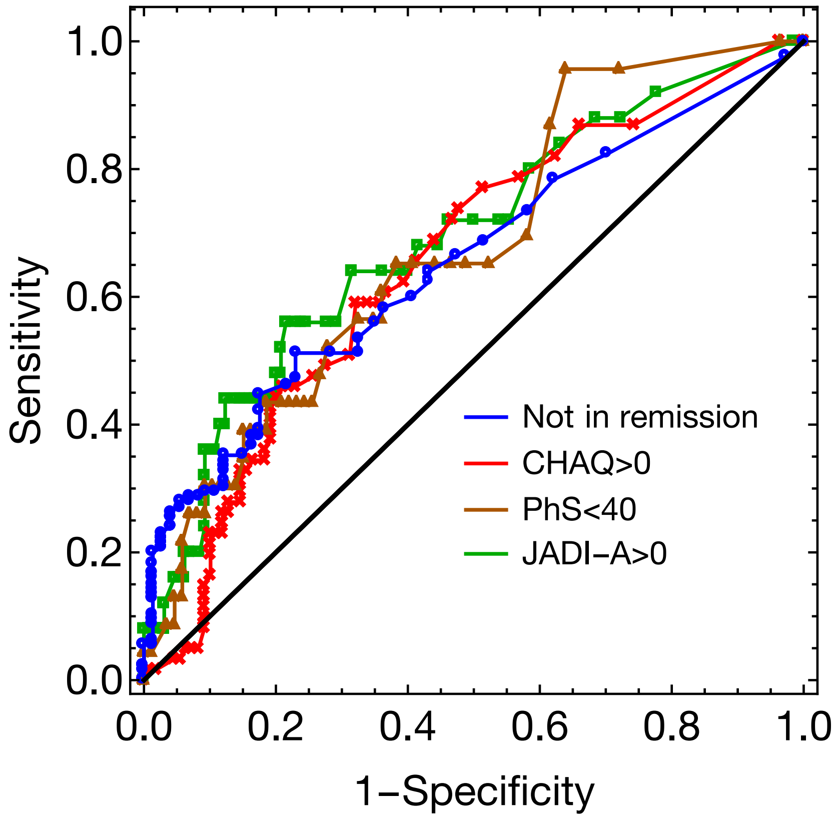

Figure S2 Receiver operating characteristics (ROC) curves for a test in the Nordic JIA cohort of the prediction model for severe disease course by Guzman et al. The area under the curve (AUC) values were 0.69 for non-achievement of remission off medication, 0.68 for Childhood Health Assessment Questionnaire (CHAQ>0), 0.69 for Physical Summary Score (PhS<40), and 0.71 for joint damage (JADI-A>0).
